# Rapid Assessment of SARS-CoV-2 Transmission Risk for Fecally Contaminated River Water

**DOI:** 10.1021/acsestwater.0c00246

**Published:** 2021-02-22

**Authors:** Jamie D. Shutler, Krzysztof Zaraska, Thomas Holding, Monika Machnik, Kiranmai Uppuluri, Ian G. C. Ashton, Łukasz Migdał, Ravinder S. Dahiya

**Affiliations:** † University of Exeter, Penryn Campus, Penryn TR10 9FE, U.K.; ‡ Łukasiewicz-Institute of Electron Technology, 01-919 Warsaw, Poland; § University of Agriculture in Kraków, 30-239 Kraków, Poland; ∥ Bendable Electronics and Sensing Technologies (BEST) Group, University of Glasgow, Glasgow G12 8QQ, U.K.

**Keywords:** dilution, water transmission, fecal−oral, sewage, SARS-CoV-2

## Abstract

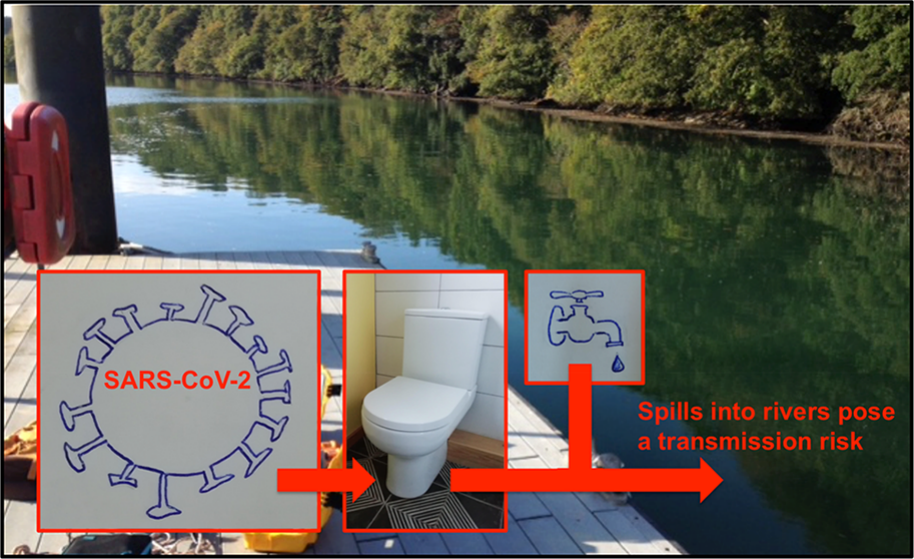

Following the outbreak of severe acute respiratory syndrome coronavirus (SARS-CoV-2), airborne water droplets have been identified as the main transmission route. Identifying and breaking all viable transmission routes are critical to stop future outbreaks, and the potential of transmission by water has been highlighted. By modifying established approaches, we provide a method for the rapid assessment of the risk of transmission posed by fecally contaminated river water and give example results for 39 countries. The country relative risk of transmission posed by fecally contaminated river water is related to the environment and the populations’ infection rate and water usage. On the basis of in vitro data and using temperature as the primary controller of survival, we then demonstrate how viral loads likely decrease after a spill. These methods using readily available data suggest that sewage spills into rivers within countries with high infection rates could provide infectious doses of >40 copies per 100 mL of water. The approach, implemented in the supplementary spreadsheet, can provide a fast estimate of the upper and lower viral load ranges following a riverine spill. The results enable evidence-based research recommendations for wastewater epidemiology and could be used to evaluate the significance of fecal–oral transmission within freshwater systems.

## Abstract 2

Sewage spills pose a risk for SARS-CoV-2 fecal−oral transmission; risk is governed by population dynamics, dilution, and the environment.

## 1 Introduction

The outbreak of the severe acute respiratory syndrome coronavirus (SARS-CoV-2) began in Wuhan Province, China, in December 2019 and, as of January 2021, has now spread throughout the world with more than 99 million confirmed cases globally within 223 countries, areas, or territories. Water droplets originating from individuals infected by SARS-CoV-2 are considered a major transmission pathway for infection,^1^ and the virus has been shown to remain stable in a saline solution^2^ and under varying environmental conditions.^3^ Viral shedding in feces of viable SARS-CoV-2 virus has been documented (e.g., ref (4)), and SARS-CoV-2 ribonucleic acid (RNA) has been detected in the shed feces of both symptomatic and asymptomatic children and adults (e.g., ref (5)), with potentially 43% of infections being asymptomatic and unreported.^6^

Human viral pathogens that can be transmitted by water that pose moderate to high health significance as defined by the World Health Organization (WHO) include adenovirus, astrovirus, hepatitis A and E, rotavirus, norovirus, and other enteroviruses. The survival of the large family of coronaviruses in water systems has been highlighted,^7−12^ and SARS-CoV-2 viral RNA loads within untreated wastewater, consistent with population infection rates, have been identified.^13^ In addition to the presence of viral RNA, the presence of viable virus must also be demonstrated. While evidence for SARS-CoV-2 is limited, other human coronaviruses are documented to survive in wastewater,^14^ with a colder water temperature likely to increase survival considerably.^3^ Collectively, this evidence suggests that SARS-CoV-2 virus could survive within water and the viral loads within untreated sewage effluent are likely high in countries with high infection rates, a portion of which is viable virus. Therefore, water contaminated with sewage provides a potential fecal–oral transmission route.^15,8,12,11^ However, identifying evidence of the presence of the virus and particularly infectivity within water systems (sewage from urban environments, within sewage treatment works, treated wastewater, or natural water bodies after sewage spills) requires more investigation.^12,8^ SARS-CoV-2 has been detected in river water of a country with low sanitation methods^16^ and in treatment plant wastewater,^13,8^ and contamination from combined sewer outflow events poses a particularly high risk.^17^ However, data on infectivity are limited^8^ and have so far been negative.^12^ These negative results are from limited sample sizes and locations; hence, a precautionary approach to the infection risk posed by sewage spills is advocated.^12^

Sewage can directly enter natural water systems due to combined sewer overflow events and sewage exfiltration from pipes (e.g., ref (18)), unexpected failure of water treatment systems, or a complete lack of water treatment infrastructure (e.g., ref (16)). For example, during the current pandemic large sewage spills, flooding dwellings and community spaces, have occurred in the United States (Georgia, Florida, and New York) and Spain (Andalucia), while temporary settlements (e.g., shanty towns, favelas, or bustees) and refugee camps are less likely to have safe sanitation systems. In these settings, this water system pathway could enable transmission of the virus to humans or other susceptible animals via water ingestion, aerosol generation, or filtering of water during feeding. It is also clear that conventional wastewater treatment methods only partially remove SARS-CoVs, highlighting the need for wastewater risk assessments and methods tailored to SARS-CoV-2.^19^

The highly skewed distribution of infected patient viral loads observed^20^ includes the effects of superspreaders, where single individuals can be responsible for a majority of the viral load. This viral distribution means that sewage originating from populations that contain superspreaders will contain very high viral loads, even though the majority of the population contributes relatively low viral loads. This issue further highlights the need for tools to identify the risk of transmission and infection associated with sewage spills, particularly within large populated areas.

Considering the information presented above, we modify established pollution analysis methods^21^ to estimate the viral concentration in rivers following a spill, allowing the relative risk of transmission posed to humans by contaminated waterways to be calculated for 39 countries. Similar methods have been successfully used to study labile organics from sewage and thermal pollution within global river systems.^22^ Modeling the survivability of SARS-CoV-2 in a solution using published in vitro study data^3^ then enables the temporal reduction in viral load and risk over multiple days to be estimated. The results using infection numbers on May 3, 2020, for 21 countries, where inland water temperatures were available, identify viable waterborne virus concentrations that could be in the water if a spill had occurred. The method provides a fast way to assess the risk of transmission associated with sewage spills through the use of easily available population, infection rate, and environmental data. The implications of the analysis from the example countrywide data for transmission of the waterborne virus to humans and animals are discussed. Recommendations for advancing this work and the limitations of the method are also given.

## 2 Methods

### 2.1 Input Data

All dilution factors (DFs) were taken from ref (21). The numbers of confirmed cases, fatalities, and recovered cases were taken from the Worldometers Web site (https://www.worldometers.info/). The long-term statistical mean global inland lake water temperature climatology was constructed using the 0.05° × 0.05° daily resolution GloboLakes version 4 data set^23^ that covers the period of 1996–2016. Mean, lower, middle, and upper percentile temperatures were calculated for each calendar month across all years producing 12 monthly data sets with a 0.05° × 0.05° gridded resolution. The water temperature uncertainty terms were propagated into the monthly data set by assuming random errors were independent and normally distributed, and using standard uncertainty propagation methods. The resulting uncertainty term combines the original uncertainty in measurement and optimum interpolation with the spatial/temporal uncertainty of the resampled monthly average, for each grid cell. These uncertainty data were used to confirm the integrity of the resampled data set.

### 2.2 Relative Risk of Transmission from Wastewater Spills

The between-country relative risk of SARS-CoV-2 transmission from wastewater systems (*H*_c_) is calculated from the per capita volume of wastewater and dilution factor for each country. Modified versions of eqs 1 and 2 from ref (21) are first used:
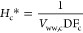
1where *V*_ww,c_ is the per capita daily volume of domestic water usage for each country c and DF_c_ is the dilution factor downloaded from ref (21) (supplemental Table 1 and supplemental Table 2, respectively). The DF_c_ is dependent on the river flow and the total domestic wastewater effluent within each country. Normalizing *H*_c_* for each country by the maximum value (*H*_max_*) provides the between-country relative risk of waterborne transmission due to the viral load in an average river following a sewage effluent spill from an urban system. Note that the maximum *H*_c_* value is calculated using the country median dilution factors (DF_c_) as some countries exhibit non-normal dilution distributions. *H*_c_ is therefore defined as
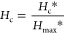
2where *H*_max_* is the maximum *H*_c_* value from all countries being considered.

### 2.3 Estimating the Number of Infectious Viruses Resulting from a Sewage Spill

The number of infectious viruses in the water system as a result of a wastewater spill or leak is calculated by multiplying *H*_c_ by the number of infectious viruses in feces generated per capita, *C*_inf,c_. *C*_inf,c_ is first calculated using
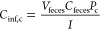
3where *V*_feces_ is the volume of feces generated (liters per capita per day), *C*_feces_ is the number of viral RNA copies in fecal matter (per liter), *P*_c_ is the proportion (prevalence) of the population of country c that has active infections, and *I* is the ratio of viral RNA copies to viable (infectious) virus.

To calculate *C*_feces_, we assumed a log-normal distribution and calculated the expected value using the mean and standard deviation from ref (20) using the standard equation
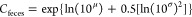
4where μ is the sample mean and σ is the sample standard deviation of the log-normal distribution. Jones et al.^20^ state that the μ of the distribution is 5.22 log_10_ copies mL^–1^ and σ = 1.86 log_10_ copies mL^–1^, which results in an expected *C*_feces_ in feces of 1595.9 million copies mL^–1^. Note that the data of Jones et al.^20^ are used here because of the large sample size and the reporting of fitted distributions and distribution parameters for the mean and standard deviation, making integration into our model straightforward. These data come from sampled respiratory fluid, not fecal matter; however, the distributions of viral particle concentrations in respiratory and fecal samples appear to overlap considerably (Figure S1^5,24^). As noted in refs (5) and (20), the viral load follows a heavy-tailed distribution with the majority of patients shedding ∼10^5^ copies ml^–1^ but some having viral loads as high as 10^12^ copies mL^–1^. This results in the superspreader problem in which a tiny proportion of the infected population can become responsible for contributing a majority of viral load to the wastewater. For a large infected population, this approach allows robust statistical modeling of viral load. However, in the case of smaller communities with a low number of infections, the actual viral load could be severely underestimated if a superspreader is present within the population. *V*_feces_ is the mean daily volume of feces generated per person (0.149 kg, from Table 3 of ref (25), and assuming feces has a density approximately equal to that of water).^26^ Note we used the “high-income country” value from ref (25) because the data^20^ that we use to estimate *C*_feces_ were measured from samples collected in Germany. Assuming that the total number of viral particles shed does not differ between high- and low-income countries, it therefore does not matter whether the *V*_feces_ and *C*_feces_ data come from high- or low-income countries, but the values used should be mutually consistent. The prevalence data, *P*_c_, were calculated by subtracting the number of recovered and the number of fatalities from the number of confirmed cases from the Worldometer Web site to obtain the number of active cases and then dividing by the country population:
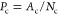
5where *A*_c_ is the number of active cases and *N*_c_ is the population of the country.

The polymerase chain reaction (PCR) method (used in ref (20)) does not distinguish between infectious virus and damaged/destroyed non-infectious virus. Therefore, to estimate the number of viable (infectious) virus copies, we used literature about the ratio of infectious adenovirus copies to genome copies in raw sewage^27^ and wastewater discharged into rivers.^28^ These estimates varied over 4 orders of magnitude, and therefore, we selected high (10^–1^), medium (10^–2^), and low (10^–3^) estimates to represent the large range of uncertainty in this ratio. These equate to 10%, 1%, and 0.1% proportions, respectively, of viable virus within the total viral genome counts, and this approach is consistent with that used in a previous SARS-CoV-2 study.^29^

The expected number of infectious viruses resulting from a sewage spill into a river or lake for a given country can therefore be calculated as
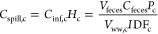
6

*C*_spill,c_ was estimated for May 3, 2020, for 21 countries that (i) contain large inland surface water bodies and so water temperature data were accessible and (ii) were known to rely upon surface reservoirs or lakes for drinking water.^30^ The long-term statistical mean inland water temperature, needed to calculate virus survivability, was therefore calculated from a climate quality global lake temperature data set (see section 2.1). The temperature values extracted for each country were the countrywide interquartile lake temperatures determined using a rectangular box as a simplified country outline. The dilution factors reported in ref (21) can vary by several orders of magnitude and were deemed to provide the major source of uncertainty in the calculation. Therefore, the *C*_spill,c_ viral loadings given by the 25th percentile dilution, 50th percentile (median) dilution, and 75th percentile dilution values are all presented. With high, medium, and low estimates for *I*, this results in nine estimates of *C*_spill_ for each country.

### 2.4 Temperature-Dependent Survival

Temperature is an important variable for determining the survival of SARS-CoVs^19^ and the stability of SARS-CoV-2 in water.^17^ As reported in ref (3), the virus concentration in solution follows an exponential decay, with its half-life increasing with a decrease in temperature, and the pH control of half-life is very small over the pH range of 3–10 (which encompasses the range found in natural freshwater and marine systems). On the basis of the in vitro data presented in ref (3), the following empirical model was derived to describe the virus concentration reduction factor due to the temperature-dependent die-off:
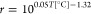
7
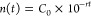
8where *C*_0_ is the initial virus concentration (copies per milliliter), *n*(*t*) is the virus concentration after time *t* (days), and *r* is the 24 h survival factor due to temperature *T*-driven die-off. This model fit to the in vitro data gives a root-mean-square difference (RMSD) of ±1% for water at 4 °C, which increases to ±7.5% at 22 °C. This parametrization is used as a proxy for survival within freshwater. Upon consideration of temperature-controlled survival in the wastewater system, *C*_spill,c_ becomes the value used for the initial viral load *C*_0_ following a sewage spill.

### 2.5 Uncertainty Analyses

A combined uncertainty budget for eq 6 was calculated using standard uncertainty propagation methods^31^ and estimates of the uncertainties of each input data set; uncertainty values are from published sources or reasonable estimates. Uncertainty components (and their values) were domestic water usage (*V*_ww,c_, ±10%), population size (*N*_c_, ±1%), number of active cases (*A*_c_, ±20%), and volume of feces generated per capita per day (*V*_feces_, 0.149 ± 0.095 L, from Table 3 of ref (25)). The analytical uncertainty for the mean number of viral genome copies in feces (*C*_feces_, 1595.9 million copies mL^–1^) and density of feces were not included in the uncertainty analysis as no values, or applicable exemplar values, could be identified or estimated from the literature. The complete analysis resulted in a combined uncertainty budget of ±68% copies mL^–1^. This result is consistent with the published uncertainty of ±50%^32^ that was experimentally determined for the original method of Keller et al. (which is the basis for eqs 1 and 3). It is important to note that the ±68% value (which describes the uncertainty for the full method, eqs 1–6) does not include uncertainty in the dilution factors or the ratio of viral genome copies to infectious viruses. Instead, and as described above, the results are calculated for a range of dilution factors, and the *C*_inf_ calculation was repeated for high, medium, and low values (i.e., the 10%, 1%, and 0.1% proportions, respectively). This approach helps to illustrate the sensitivity of the approach to these two parameters (DF and *I*). For the temperature-controlled survival calculation, eqs 7 and 8 were calculated for the 25th, 50th (median), and 75th percentiles of surface freshwater temperature to quantify their sensitivity to the regional temperature variations.

## 3 Results and Discussion

**Figure 1:**
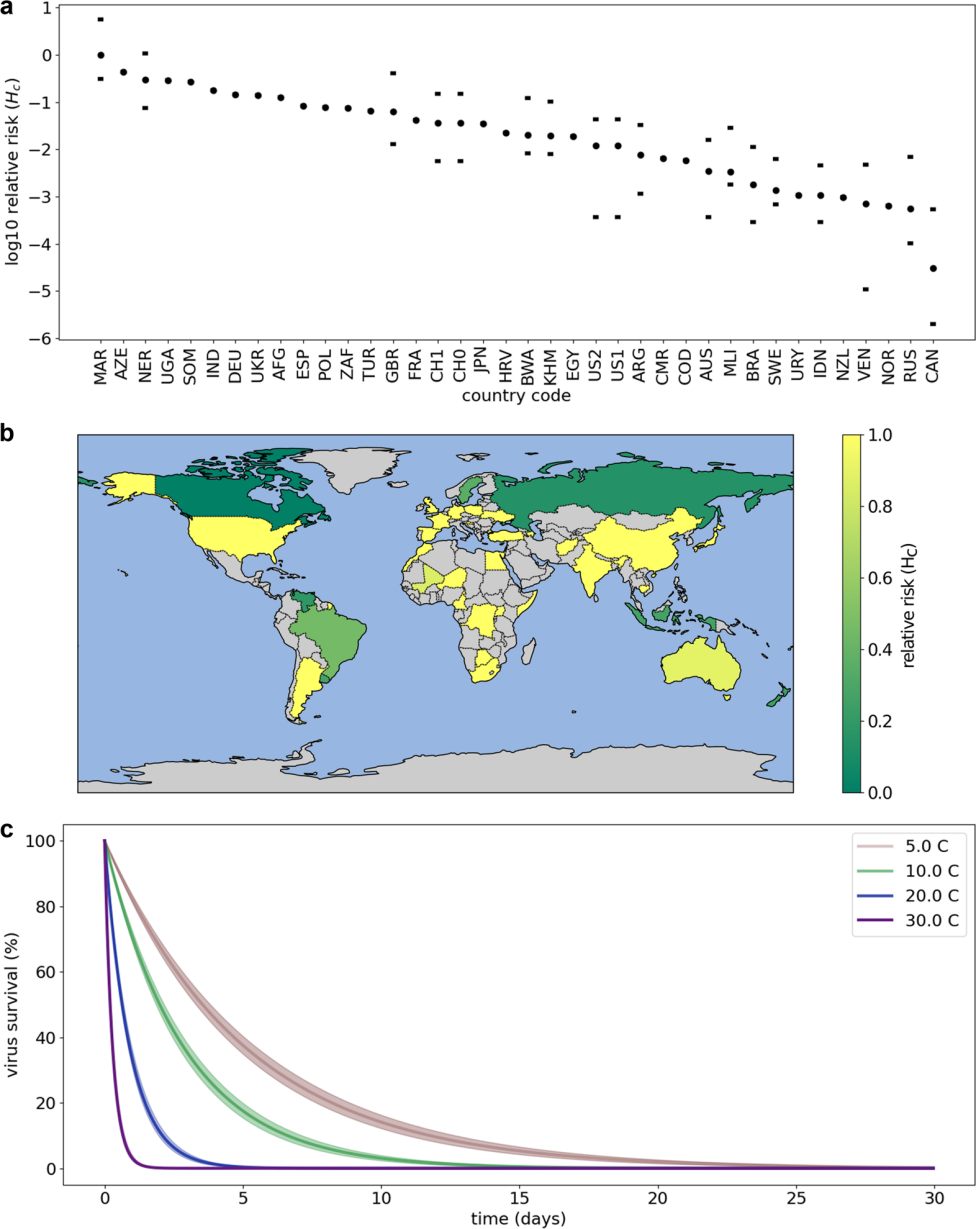
Virus survivability within freshwater and normalized country comparable risk of transmission (termed relative risk, *H*_c_) associated with sewage spills after dilution by domestic water usage and river flow (results for 39 countries) . (a) Log_10_ relative risk (*H*_c_) covering the range of 0.001–1.0. Circles are median values, and horizontal lines show the 25th and 75th percentiles due to dilution factors from ref (21). (b) Countries where relative risk, *H*_c_, has been calculated with relative risk as a linear scale. Gray signifies a country not included. (c) Modeled temperature survivability. The root-mean-square difference (RMSD) for each curve is given by the shaded areas.

The relative risk (which is the normalized country comparable risk associated with a sewage spill after dilution) shown in Figure 1a,b is dependent upon total domestic wastewater effluent (which helps to dilute the virus at the input to the system) and riverine flow (which dilutes the virus once in the river). The latter is dependent upon geographical location, geographical relief, and weather. Countries with the lowest relative risk are those with both high domestic wastewater effluent and high riverine dilution (e.g., Canada, Norway, and Venezuela). The highest relative risk results from a combination of low to medium domestic wastewater effluent and low dilution (e.g., Morocco, Spain, and Germany). Exponential temperature-driven survivability reveals that the virus can remain stable in a solution for at least 25 days (Figure 1c; at 4 °C, it can take 25 days for a 10-fold reduction).

**Figure 2:**
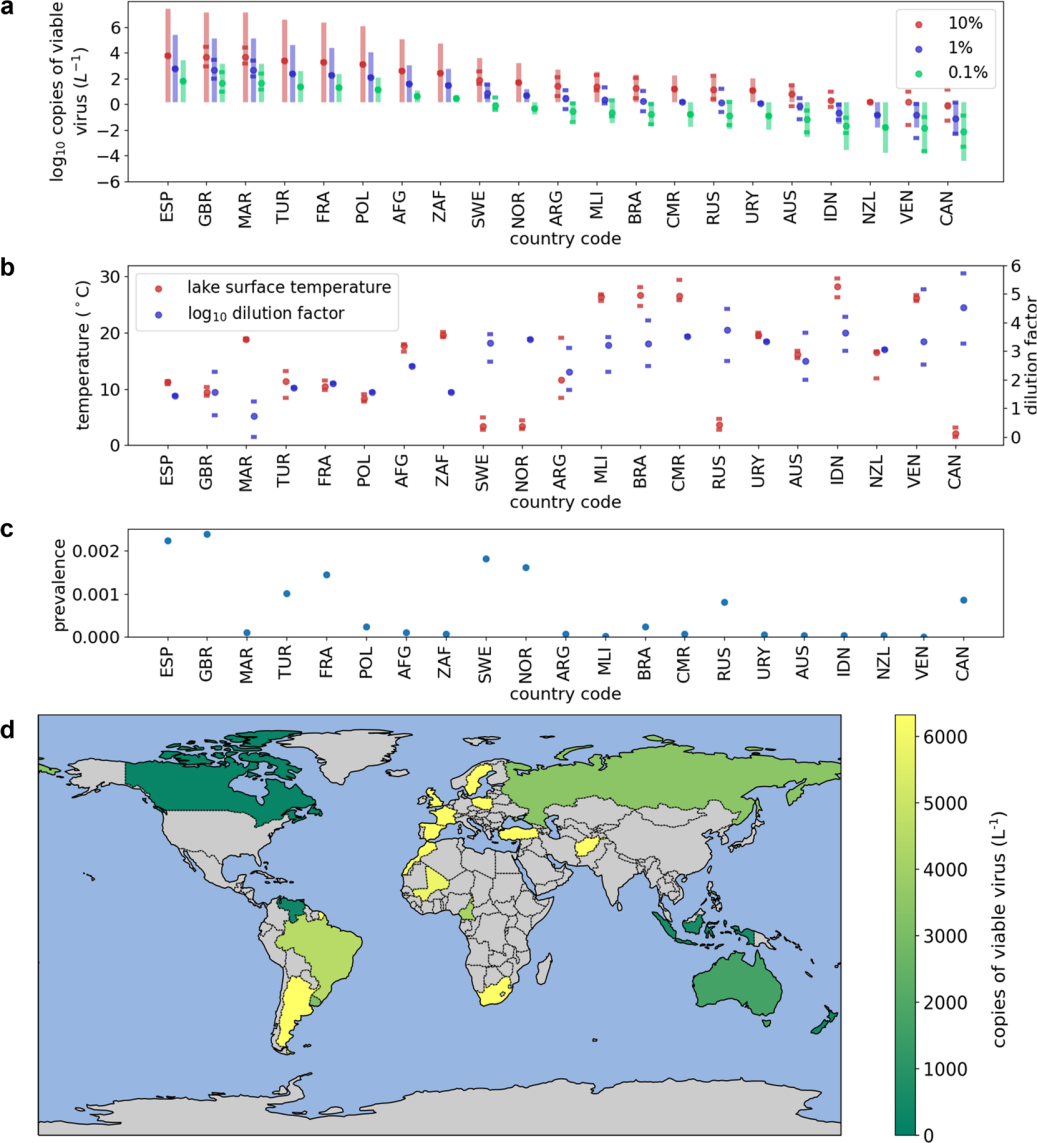
Estimate of absolute viable viral concentrations within inland waters on May 3, 2020, for 21 countries assuming a sewage spill has occurred. Circles are medians, and horizontal lines shows the 25th and 75th percentiles due to dilution factors. (a) Absolute viable viral concentrations in log_10_ copies. The shaded uncertainty bars are ±68% copies L^–1^. Results are shown for three possible ratios of viable virus to viral genome copies (10%, 1%, and 0.1%). (b) Lake water temperature and dilution. (c) Virus prevalence. (d) Countries where viable viral loads have been calculated. Gray signifies a country not included, as inland water temperature data were not easily discernible; viral concentrations are presented as a linear scale in copies of viable virus per liter.

Translating these results to the proportion of the population infected within 21 countries on May 3, 2020, identifies the estimated upper and lower limits of viable waterborne virus concentration within the first 24 h, assuming that a spill occurred (Figure 2; the uncertainty in the viable virus concentration is ±68% copies L^–1^). This analysis was limited to these 21 countries as only these had easily discernible inland water temperature data. Absolute concentrations are higher and will exist longer within countries with a combination of higher relative risk, colder water, and high population infection rates. A person in the three countries with the highest concentrations (Spain, U.K., and Morocco) who within the first 24 h of a spill ingests 100 mL of the contaminated water could be exposed to a total dose of 46–3080 copies, where the large range is driven by the chosen ratio of viral RNA copies to viable (infectious) virus (*I*) and dilution (DF) (Table 1).

**Table 1:** Viable Virus Concentrations for the Three Countries for May 3, 2020, Assuming a Spill Occurred^a^

country	^Ψ^*I* = 0.1%, median DF (copies L^–1^)	*I = 1%, median DF (copies L^–1^)	^**$**^*I* = 10%, median DF (copies L^–1^)	^&^*I* = 10%, low DF (copies L^–1^)	100 mL dose for case $ and total range (copies)	24 h survival (%)	48 h survival (%)
Spain (SPA)	63	632	6325	6325	633 (63^Ψ^–633^&^)	67 (66–68)	45 (44–47)
U.K. (GBR)	47	468	4682	30792	468 (47^Ψ^–3080^&^)	72 (70–74)	52 (49–55)
Morocco (MAR)	46	459	4595	25255	459 (46^Ψ^–2526^&^)	38 (38–39)	15 (14–15)

a Median dilutions (DFs) along with low^**Ψ**^, middle*, and high^$^ viable:unviable viral ratios (*I*) are given to provide a reasonable range of concentrations within the first 24 h. ^&^Low DF (lower 25th percentile) and high *I* results enable the upper range of concentrations to be estimated. Viral survival rates after 24 and 48 h show how the viable viral concentrations decrease due to temperature-driven die-off. Survival is for the median lake temperature, and the ranges are the 25th and 75th temperature percentiles.

The combination of Figures 1c and 2a can be used to understand the viable virus concentration after the first 24 h. The water temperature-controlled virus survivability means that concentrations will likely decrease quickly in Morocco within 24 h of a spill, whereas the concentrations remain for longer periods in Spain and the U.K., where water temperatures are lower (Table 1 and Figure 2b).

### 3.1 Placing the Dosage Results in Context

One hundred milliliters is the equivalent of one or two mouthfuls of water, and swimmers can swallow up to 280 mL in a 45 min swim.^33^ The dose of SARS-CoV-2 virus needed for infection is not known. Nikitin et al.^34^ provided 10^3^ copies for influenza, and the infectious dose for SARS-CoV-2 is likely significantly lower because ref (35) ranks influenza as “very high infective dose” and SARS-CoV-2 as “low”. Therefore, the example dose highlighted in the results of 46–3080 copies within two mouthfuls of water would appear to be worrying. It is possible that inhaling aerosols originating from these waters could pose a greater transmission risk, as the aerosols could pass back and forth multiple times through the respiratory system as the person breathes in and out, increasing the chances of an interaction with the abundant respiratory angiotensin-converting enzyme 2 (ACE2) receptors and allowing infection. Whereas ingested water will generally go down the throat into the stomach providing fewer opportunities to interact, or delaying their interaction, with any ACE2 receptors, but this is unconfirmed.

### 3.2 Limitations of the Methods and Areas of Future Work

This work demonstrates a methodology for rapidly assessing the potential countrywide transmission risk posed by sewage spills into river systems. However, several knowledge gaps need to be addressed, particularly if these methods are to become more applicable to regional or local scales (e.g., for individual wastewater treatment plants or specific recreational bodies of water).

The analysis is based on countrywide infection rates and dilution factors, whereas in practice, these will vary spatially within countries. Pathogen prevalence will usually have strong spatial structuring and variation, so the use of regional infection rates will likely improve any results. Some countries exhibit high temporal variability in dilution factors (e.g., India^21^), highlighting the need for a higher-spatial resolution analysis. Notwithstanding this, the measured wastewater viral counts in Paris on April 9 were 3.1 × 10^6^ genome copies L^–1^ with 82000 active cases,^36^ whereas using our (albeit country specific) method gives an estimate of 1.3 × 10^6^ genome copies L^–1^, which is within the correct order of magnitude. These calculations used the same number of active cases and do not incorporate dilution by natural water systems, so the values are directly comparable. This independent validation data point implies an uncertainty of ±58% for this part of the calculation, which is slightly larger, but still consistent, with the ±50% uncertainty that was experimentally determined for this part of the calculation^32^ and therefore provides further confidence in our methodology.

SARS-CoV-2 is still a relatively new virus, and more data are needed to understand its basic behavior. This includes the need to quantify the infective dose, the number of viable virus particles in feces, and larger data sets to study its viability in water systems.^7^ The detection of virus RNA in the aquatic environment^9^ does not necessarily translate into the presence of viable virus. To estimate the number of viable (infectious) virus copies, the proportion of infectious viruses in sewage must be known. The presence of infectious viruses in stool samples has been demonstrated,^4^ but there is a lack of quantitative data about this ratio for SARS-CoV-2 in stool. We instead used literature on the number of infectious adenovirus copies in sewage (e.g., ref (27)) and wastewater discharge into rivers^28^ to select high (10^–1^), medium (10^–2^), and low (10^–3^) estimates for the ratio of infectious viruses to genome copies. We note that adenoviruses are known to be particularly resilient and therefore likely to represent an upper estimate, but also that our selected range is consistent with the 10^–3^ value used elsewhere for assessing viral risk in water systems (e.g., ref (33)), including one assessment for the risk of transmission of SARS-CoV-2 to wastewater workers.^29^ In this study, the SARS-CoV-2 survivability model is based on in vitro data (using artificial saline buffer) and assumes that the water temperature is the dominant (first-order) controller. The same in vitro data suggest that variations in pH expected within riverine water have a minimal effect on survival, but the combined analysis ignores any ultraviolet, biological, or bacterial influences and deactivation by detergent. The analysis therefore provides the upper range of perceivable transmission risk, rather than providing a precise value of the absolute risk, and the survivability based on temperature (valid for varying pH values) is provided purely as an indicator of the viral die-off.

Ultraviolet (UV, wavelengths of 280–400 nm) radiation (e.g., from sunlight) can inactivate SARS-CoV-2,^37^ although the most biologically damaging part of the spectrum (UV-B, 280–315 nm) is strongly absorbed by water, leaving the virus particles intact. In contrast, UV-A (315–400 nm) radiation can penetrate oceanic water^38^ but is less efficient at deactivating SARS-CoV-2.^39^ These results suggest that the detrimental effect of all UV radiation on virus survival appears skewed toward the short-wavelength part of the spectrum, which is less able to penetrate water. The absorption signals of fresh and marine water will differ, but clearly, penetration of the water column by UV radiation from sunlight could contribute to some virus inactivation (as highlighted in ref (40)); however, the inability of the major damaging wavelengths to penetrate the water means UV is unlikely to be a major controller of virus survival within natural water systems. More work is needed on the impact of multiwavelength UV-A radiation on virus survival and its attenuation in river water, and identifying if short bursts of high UV-A or longer radiation of lower-intensity irradiance (as found in nature) has equal impacts on virus inactivation.

### 3.3 Issues Related to Potable Water

It appears feasible that coronaviruses could enter drinking water systems particularly where low disinfection rates are applied.^11^ It is possible that SARS-CoV-2 survivability and transport within rivers could impact water supplies for drinking, washing, and cooking in countries where rivers or reservoirs are the primary drinking water sources and where large populations, with little or no sewage treatment, exist close to the water source, such as within refugee camps or shanty towns. Riverine enteric virus transport and catchment accumulation can occur for common viruses (e.g., ref (41)), and under stratified conditions, it is possible for a river plume to enter a reservoir and subsequently exit through the reservoir outlet, and therefore into the potable water supply, without mixing with the main water body.^42^ The World Health Organization guidelines state that effective chlorination disinfection for potable water occurs at residual chlorine concentrations of ≥0.5 mg L^–1^,^43^ which matches the minimum needed to deactivate SARS-CoV-1.^44^ Therefore, existing water disinfection guidance appears to be sufficient,^9^ but these will need to be followed to ensure that waterborne transmission is not possible.

### 3.4 Recommendations for Reducing the Risk of Transmission to Wildlife and Accumulation in the Environment

SARS-CoV-2 originating from untreated wastewater has been identified within seawater.^9^ Bioaccumulation of the SARS-CoV-2 virus by molluscs and other aquatic organisms may occur within contaminated estuarine waters, as other waterborne viruses, including hepatitis, norovirus, and avian influenza, are known to accumulate in bivalves (e.g., clams^45^ or oysters). Some cetaceans are susceptible to SARS-CoV-2,^46^ and coronaviruses have previously been detected in a beluga whale (whale-CoV SW1) and dolphins (cetacean coronavirus). Some may be at risk of infection if sifting or filtering large amounts of contaminated river water or sewage outflow water to extract their food (e.g., orca feeding on Chinook salmon). Collectively, these findings suggest that novel volume integrating viral detection methods, needed for use within water treatment systems,^8^ may also be needed to ensure the safety of the natural environment.

An increased level of animal foraging can occur downstream from water treatment facilities, relative to upstream, highlighting the risk of infecting susceptible riparian and semiaquatic wildlife.^47^ The need for surveillance of SARS-CoV-2 in cats has already been highlighted as an adjunct to elimination of COVID-19 in humans.^48^ It is possible that surveillance of susceptible riparian and semiaquatic wildlife known to have been exposed to, or have access to, sewage-contaminated waters could be needed as an adjunct to elimination of COVID-19 in humans.

### 3.5 Recommendations for Identifying the Significance of Fecal–Oral Transmission and Reducing Uncertainty in Risk Assessments

Clearly, there is a fundamental need to evaluate the prevalence, infectivity, and viral viability within water systems to assess the risk of transmission^7^ and to underpin wastewater epidemiology.^17^ The detection limit of the real-time polymerase chain reaction (RT-PCR) methods used to detect the presence of SARS-CoV-2 within human samples is 100 copies mL^–1^.^49^ From Table 1, the infectious viral load for Spain on May 3, 2020, would have been below the PCR detection limit at 0.06–6.33 copies mL^–1^, and 24 h later, it had decreased to 0.04–4.24 copies mL^–1^ (67% survival). These low values, driven by dilution and then temperature-driven die-off, highlight the need for concentration methods (e.g., as reviewed in ref (17)), but the identified temperature-driven die-off means that the effluent temperature history is needed, from fecal input onward, to fully interpret any PCR viral loads. The PCR result is valid for a snapshot in time, and understanding where the PCR value falls in relation to the viral survival curves in Figure 1c will place the PCR result into context. The potential die-off during sample analysis may also be important as some concentration techniques integrate samples over time.^17^ The large intercountry variations in dilution^21^ and the lack of temporal history could be one reason for the unexplained range of SARS-CoV-2 viral loads so far found in wastewater (Table 4 of ref (17)) and the inconclusive viral infectivity results. For example, if you have a virus concentration value, the detection threshold, and a temperature history, then you can calculate when you would expect the virus concentration in the sample to fall below the PCR detection thresholds using our methods. If you also have an understanding of the DF (within the water system sampled), then you can determine when the virus concentration in the water system will fall below the PCR detection thresholds. Our results suggest that only targeted wastewater sampling that considers the regional water usage, regional rainfall (collectively capturing regional dilution), regional infection rates, and the complete effluent and sample temperature history are likely to produce robust infectivity conclusions for water systems. Viral and infectivity loads measured under controlled experimentation using feces from infected individuals, where the loads are assessed at the source and then at regular intervals after dilution and over multiple days, repeated for different water temperatures, are likely needed to support the interpretation of field data. The results from such experiments and sampling strategies are needed for wastewater epidemiology. Figure 2a (which is in log_10_ copies per liter of viable virus) shows that the results presented here are highly sensitive to *I* (the ratio of viral RNA copies to viable infectious viruses, i.e., 0.1%, 1%, and 10%), so experiments to determine conclusive viral and infectivity loads would also significantly reduce the uncertainty in the methods presented herein.

With respect to identifying significant transmission routes, data are also needed to enable causation of outbreaks to be evaluated. International efforts for “track and trace” methodologies currently consider transmission from only direct or close interactions between individuals, whereas data on the potential infectious nature of the environment itself with which individuals interact are missing from these approaches. Large scale transmission or infection causality evaluation that includes data on the environmental conditions and periods where airborne transmission and water transmission are possible will allow the evaluation of the significance of each transmission route. The methods presented in this paper, applied at a regional level within countries, would provide a key data set needed for such an analysis.

## 4 Conclusions

Natural water systems are likely able to act as a transmission pathway for SARS-CoV-2, which is a threat to humans and animals. To help address this issue, this paper provides a method for the rapid assessment of the SARS-CoV-2 transmission risk posed by fecally contaminated water systems. The initial country specific analysis using the rapid assessment method suggests that public interactions with river water following wastewater spills should be minimized to reduce the risk of infection, especially in circumstances where spills coincide with aerosol generation. Applying the approach using regional data could be used (i) to quickly assess the risk of transmission to the public and wildlife posed by a spill, (ii) to identify regions where more detailed sampling and laboratory assessment are needed to accurately quantify exposure, and (iii) to identify regions that have previously been exposed to a transmission risk. The results highlight the need for volume-integrating viral detection methods to ensure the safety of the natural environment. The temporal viral survival model is given as a proxy for understanding how the risk of transmission changes with time but is based on only a primary controller (temperature) and does not include other processes that are likely to further degrade the virus. As such, it will likely overestimate the survival of any virus (i.e., underestimate viral die-off). The results presented here suggest that studies aiming to accurately identify the infectious viral loads within water and wastewater systems, which have been identified as being critical for wastewater epidemiology, need to characterize the complete temperature history of the effluent, from defecation to the end of sample analysis, if they are to produce conclusive results.

## Supporting Information Available

Supplemental text and Figure S1 (PDF)
Supplemental tables (ZIP)
