# Supplementary material for: Rapid Assessment of SARS-CoV-2 Transmission Risk for Fecally Contaminated River Water
Source: ACS ES T Water. 2021 Feb 22;1(4):949–57. doi: 10.1021/acsestwater.0c00246 (PMC7931626; doi:10.1021/acsestwater.0c00246)
Supplement: Supplementary file 1 — ew0c00246_si_001.pdf [file ew0c00246_si_001.pdf]

Rapid assessment of SARS-CoV-2 transmission risk for faecally contaminated river water

Jamie D. Shutler, Krzysztof Zaraska, Thomas Holding, Monika Machnik, Kiranmai Uppuluri, Ian Ashton, Łukasz Migdał, Ravinder Dahiya

Supplementary text

All relative risk data and calculations are provided in the supplementary file (relative_risk.xlsx). All data inputs and results for the viable viral count analysis and 24 hour and 48 hour survivability are also provided (waste_water_spill_pathogens.xlsx); this spreadsheet can be updated by a user to estimate viral counts for any day if water usage and infection rates are known. All software for all calculations, for generating the supplementary spreadsheets, along with the supplementary spreadsheets themselves are open access and can be found on the GitHub repository: https://github.com/JamieLab/CoV2-wastewater.

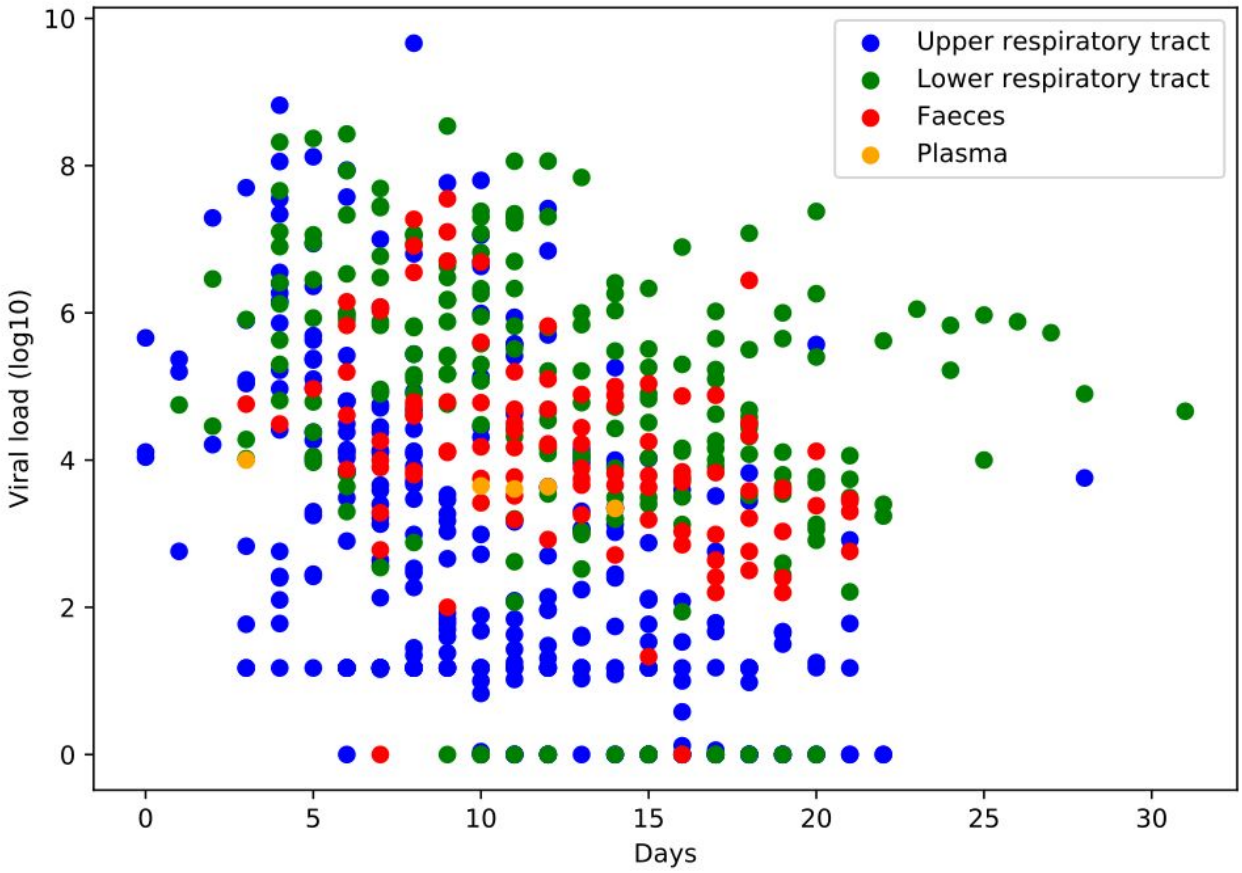

Figure S1 Viral loading for respiratory swabs and faeces plotted using the data provided within Weiss *et al.,* (2020). Data correspond to Figure 5 from Weiss *et al.,* 2020.

Reference

Weiss, A., Jellingsø, M., Sommer, M. O. A, Spatial and temporal dynamics of SARS-CoV-2 in COVID-19 patients: A systematic review and meta-analysis, *EBioMedicine*, 58, 102916, (2020) doi:10.1016/j.ebiom.2020.102916
